# Transcriptome Analysis of Cytokinin Response in Tomato Leaves

**DOI:** 10.1371/journal.pone.0055090

**Published:** 2013-01-25

**Authors:** Xiuling Shi, Sarika Gupta, Ingrid E. Lindquist, Connor T. Cameron, Joann Mudge, Aaron M. Rashotte

**Affiliations:** 1 Department of Biological Sciences, Auburn University, Auburn, Alabama, United States of America; 2 National Center for Genome Resources, Santa Fe, New Mexico, United States of America; Volcani Center, Israel

## Abstract

Tomato is one of the most economically and agriculturally important Solanaceous species and vegetable crops, serving as a model for examination of fruit biology and compound leaf development. Cytokinin is a plant hormone linked to the control of leaf development and is known to regulate a wide range of genes including many transcription factors. Currently there is little known of the leaf transcriptome in tomato and how it might be regulated by cytokinin. We employ high throughput mRNA sequencing technology and bioinformatic methodologies to robustly analyze cytokinin regulated tomato leaf transcriptomes. Leaf samples of two ages, 13d and 35d were treated with cytokinin or the solvent vehicle control dimethyl sulfoxide (DMSO) for 2 h or 24 h, after which RNA was extracted for sequencing. To confirm the accuracy of RNA sequencing results, we performed qPCR analysis of select transcripts identified as cytokinin regulated by the RNA sequencing approach. The resulting data provide the first hormone transcriptome analysis of leaves in tomato. Specifically we identified several previously untested tomato orthologs of cytokinin-related genes as well as numerous novel cytokinin-regulated transcripts in tomato leaves. Principal component analysis of the data indicates that length of cytokinin treatment and plant age are the major factors responsible for changes in transcripts observed in this study. Two hour cytokinin treatment showed a more robust transcript response indicated by both greater fold change of induced transcripts and the induction of twice as many cytokinin-related genes involved in signaling, metabolism, and transport in young vs. older leaves. This difference in transcriptome response in younger vs. older leaves was also found to a lesser extent with an extended (24 h) cytokinin treatment. Overall data presented here provides a solid foundation for future study of cytokinin and cytokinin regulated genes involved in compound leaf development or other developmental processes in tomato.

## Introduction

Cytokinins are plant hormones that occur naturally as N6-substituted adenine derivatives. Over 50 years of study has implicated this class of hormones in many aspects of plant growth and development, including de-etiolation, chloroplast differentiation, apical dominance, and leaf senescence [1], [2]. They have also been shown to regulate leaf development and stress response [3]–[5]. The cytokinin signaling pathway has been determined to be composed of cytokinin receptors (histidine kinases; HKs), signaling mediator histidine containing phosphotransfer proteins (HPts), and response regulators (RRs). It has been established along with a branch pathway that requires the HKs, HPts, and cytokinin response factors (CRFs) [5]–[7]. There are two major classes of response regulators- type-A RRs and type-B RRs. Type-A RRs are primary cytokinin response genes that are rapidly induced by cytokinin and are negative regulators of cytokinin signaling which can be activated by transcriptional activator, type-B RRs [5], [8]–[11]. In addition to the cytokinin signaling components, major cytokinin metabolic genes have been identified, including isopentenyltransferases (IPTs) responsible for cytokinin biosynthesis and cytokinin oxidases/dehydrogenases (CKXs) involved in oxidative degradation of cytokinin [12]–[14]. Some *CKX* genes are up-regulated by cytokinin whereas *IPT* genes are repressed [3], [9], [12], [15].

The various roles played by cytokinin in plant growth and development have led to efforts of genome-wide analyses of cytokinin regulated gene expression in several species like Arabidopsis and rice and clearly show that a wide range of genes are transcriptionally regulated by cytokinin [3], [6], [9], [10], [15]–[18]. One class of genes regulated by cytokinins encodes transcription factors that play vital roles in plant growth and development [3], [6], [9], [16], [17]. These findings were widely supported by genetic and molecular studies. In Arabidopsis, cytokinin was shown to up-regulate *SHOOT MERISTEMLESS* (*STM*), a member of the class I KNOX transcription factors [19]; overexpression of *STM* dramatically activate cytokinin biosynthesis gene *AtIPT7*, indicating that KNOXI function in meristem maintenance is mediated by activation of cytokinin biosynthesis [20]. Cytokinin is also known to induce *Cytokinin Response Factor* (*CRF*) genes that have been shown to be involved in or expressed during cotyledon and leaf development [6], [7].

Although some transcriptome data are available for tomato, most of it is focused on fruit biology, defense response, or other aspects not related to cytokinin or leaves [21]–[25]. In fact, very little is known about the cytokinin regulation of genes in tomato. The advent of next-generation sequencing technologies has provided powerful means to perform effective and accurate analyses of transcriptomes and genomes [26]–[29]. RNA-sequencing (RNA-Seq) has been proven to be a simpler and more powerful approach to quantifying expression at a transcriptome level, especially in species like tomato where existing microarrays cover only about a third of all genes in the genome [27]–[29]. Here we used RNA-seq to perform the first transcriptome analysis of cytokinin response in tomato leaves and one of the few conducted in species other than Arabidopsis and rice. We examined genome-wide gene expression in response to cytokinin in 13d and 35d old tomato leaves detecting 28,606 unique transcripts and more than one thousand that showed a response (at least 2.5 log2 fold change) to cytokinin in various samples. Among these cytokinin responsive transcripts were previously un-examined tomato orthologs of cytokinin regulated genes from other species, such as cytokinin oxidases, type-A response regulators, and cytokinin receptors. Additionally, we identified several novel cytokinin regulated genes, including a Xanthine/uracil permease family protein and a Cytochrome P450 with abscisic acid 8′-hydroxylase activity that are both highly induced by cytokinin. This study generated the first complete transcriptome analysis of cytokinin in tomato leaves providing valuable data for identifying cytokinin regulated genes that are involved in leaf developmental processes.

## Results and Discussion

### Transcriptome Analysis

In order to conduct a full transcriptome analysis of cytokinin response in tomato leaves of different ages we assembled a custom transcriptome reference and assessed differential expression from paired-end (2×50 bp) and singleton (1×54 bp) Illumina RNA sequences. To obtain a broader sampling of transcripts found in leaf tissue at a single plant age, tomato leaves of two different ages, 13d and 35d, were treated with exogenous cytokinin, 5 µM Benzyl Adenine (BA) or the solvent vehicle control dimethyl sulfoxide (DMSO) for 2 h or 24 h, after which RNA was isolated for sequencing. The messenger RNA was isolated via polyA selection and constructed into paired-end sequencing libraries with the TruSeq RNA sample preparation protocol from Illumina (San Diego, CA). RNA sequencing was performed on the Illumina HiSeq 2000 platform and Illumina GAIIX platform yielding an average of 18 million high-quality reads per sample (Table 1). In total, 131,158,386 2×50 bp and 60,180,592 1×54 bp reads were sequenced, resulting in over 16.4 Gbps of data. Paired-end sequences from all samples were pooled together to construct a *de novo* tomato leaf transcriptome assembly (see methods for details). The final assembly contained 28,606 synthetic ESTs and was used as a tomato leaf reference transcriptome for subsequent gene expression analyses.

**Table 1:** Transcriptome alignment and assembly statistics.

Age	13 DAYS								35 DAYS							
Treatment	(B) +CYTOKININ treatment				(D) −CYTOKININ treatment				(B) +CYTOKININ treatment				(D) -CYTOKININ treatment			
Treatment Length	2 HOURS		24 HOURS		2 HOURS		24 HOURS		2 HOURS		24 HOURS		2 HOURS		24 HOURS	
Replicate	A	B	A	B	A	B	A	B	A	B	A	B	A	B	A	B
High Quality Paired-End Reads	4,470,442	15,038,582	5,775,868	15,146,962	4,513,670	14,379,784	6,074,016	8,323,882	13,729,084	22,085,930	15,413,056	23,175,630	13,558,856	25,231,538	15,560,492	23,684,340
High Quality Single-End Reads	15,211,523	–	16,280,156	–	15,223,454	–	11,884,069	–	–	–	–	–	–	–	–	–
Uniquely aligningreads	8,567,251	7,492,978	9,389,806	8,216,386	9,090,012	7,629,681	6,903,736	6,489,199	7,346,044	13,925,649	8,027,102	13,904,662	7,871,216	14,547,760	8,324,948	14,322,386

The number of paired end and single end sequence reads and uniquely aligning reads analyzed from Illumina sequencing runs of all 16 samples are described in the table by plant age, treatment type and length. Individual sample replicates, A and B, are a pool of leaf tissues from multiple plants treated under similar conditions. The 131,158,386 2×50 bp paired-end reads were pooled and *de novo* assembled into a transcriptome assembly of 28,606 synthetic ESTs.

Gene expression was quantified as the total number of reads (paired-end and singleton reads) from each sample that uniquely aligned to the final *de novo* transcriptome reference assembly, binned by transcript using the aligner BWA (v0.5.9) [30]. An average of 9.5 million reads, from a combination of paired-end sequencing and single-end sequencing, uniquely aligned to the reference in each sample (Table 1).

**Figure 1:**
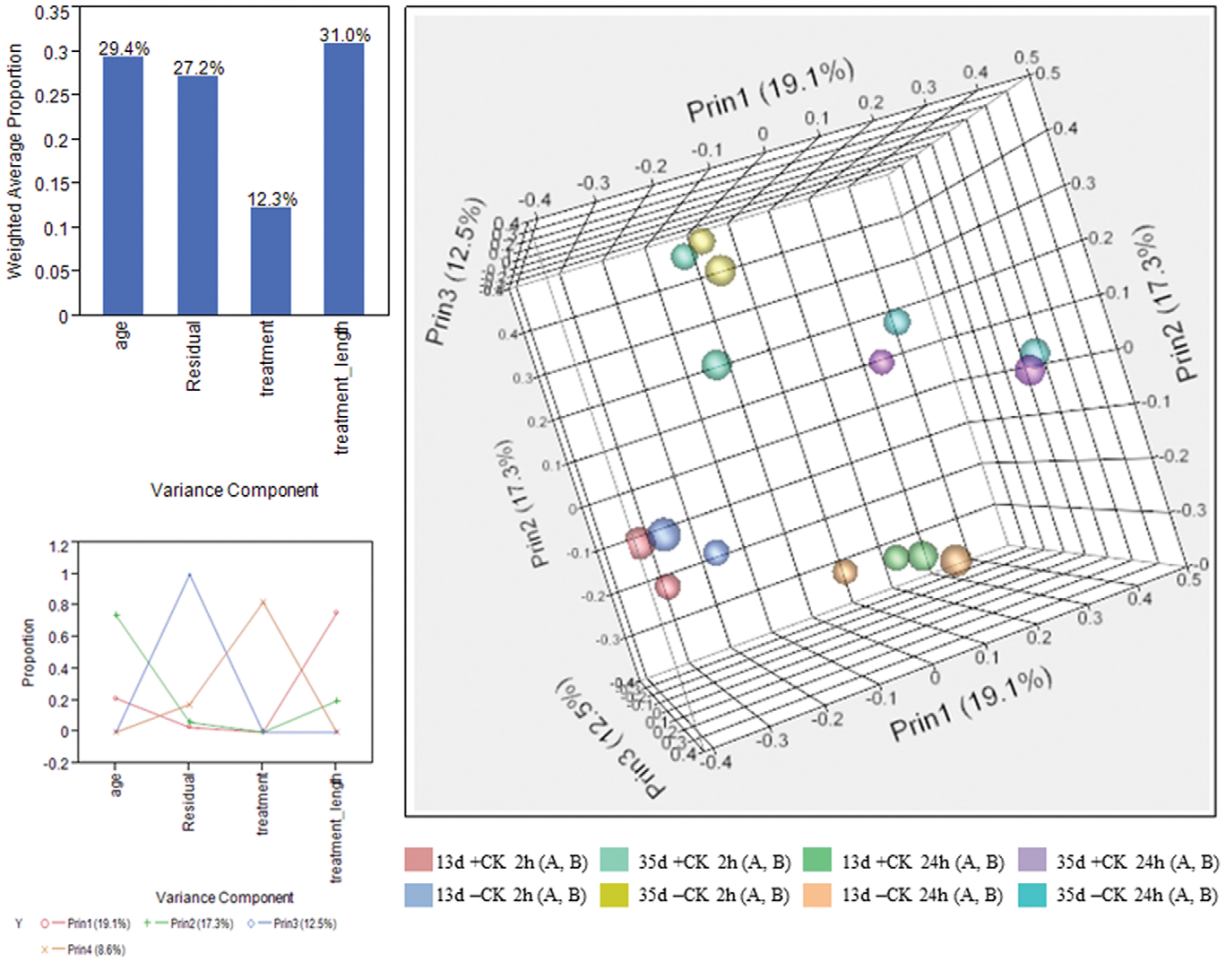
Principal Component Analysis and Variance Decomposition of Leaf Sample Variables. Principal component analysis (PCA) and variance decomposition (both as implemented in JMP Genomics 5.1) identify age of plant and cytokinin treatment length as the variables responsible for the majority of transcriptional variance, with cytokinin treatment playing a lessor role. Plots of these component principals in 2D and 3D reveal a strong clustering of individual sample replicates, A and B, as well as distinguishing age and treatment length groupings.

An initial examination of the overall dataset, normalized with the TMM strategy [31] using principal component analysis (PCA) as implemented in SAS JMP Genomics 5.1, revealed that individual replicates used in this study, A and B, clustered together indicating relatively low biological variability within sample type (Figure 1). Variance decomposition (JMP Genomics 5.1) was used to estimate the proportion of total variance attributable to the experimental variables of age, treatment and length of treatment. Together the variables plant age, cytokinin treatment, and length of treatment account for about 73% of the variance in this study, with the major factors being length of cytokinin treatment (31.0%) and plant age (29.4%) (Figure 1). Although cytokinin treatment by itself accounts for a smaller amount of the variance in this study (12.3%), together with length of treatment cytokinin clearly plays a large role in the transcript changes seen in this study.

### Cytokinin Regulation of Leaf Genes in Tomato

In order to determine the regulation of transcripts by cytokinin, differential expression analysis (see methods for details) was performed between treated and untreated samples. This revealed only a small number of different genes (8) as positively regulated by cytokinin across all treatments at a significant level (p≤0.1), although these same genes were regulated across different treatments. This includes 4 type-A cytokinin response regulators, a cytokinin receptor, a cytochrome p450– ABA oxidase, a gag polyprotein, and an unknown protein. Because this represents a small sample of the cytokinin regulated transcripts that have been identified in other species and this is the first study of cytokinin effects on tomato at a transcript level, we further investigated transcripts with high fold changes in response to cytokinin treatment that did not reach significance with DESeq. We define the transcripts that show a change of more than 2.5 log2 fold expression in response to cytokinin as cytokinin responsive genes (See Table 2, Table S4, S5). This is more than double the fold change for genes that have been identified as cytokinin regulated in other species, such as Arabidopsis and Rice using microarray analyses (set at 2 fold) [10]. With the same criteria, we also identified transcripts that are more abundant in young or older leaves (Table S6).

**Table 2:** Summary of overall transcript changes seen in major compared categories.

Categories	Early response 2 h (BA vs. DMSO)				Late response 24 h (BA vs. DMSO)				2 h (DMSO)		24 h (DMSO)	
Leaf age	13d		35d		13d		35d		13d	35d	13d	35d
Transcript changes	Induced	Repressed	Induced	Repressed	Induced	Repressed	Induced	Repressed	More abundant	More abundant	More abundant	More abundant
# of genes	60	669	14	279	97	95	91	73	926	168	198	123

The number of genes identified as cytokinin responsive (showed at least a transcript change of 2.5 log2 fold) for each of the shown comparisons is listed from the sample reads shown in Table 1. Induced (up-regulated 2.5 log2 fold vs control). Repressed (down-regulated 2.5 log2 fold vs control). More abundant (2.5 log2 fold greater than the other age sample at that treatment time).

In order to confirm the accuracy of the RNA-seq expression results, qPCR was performed to quantify the expression of select transcripts. Four DE genes and four genes identified as cytokinin responsive in 35 d plants after 24 h cytokinin treatment vs DMSO were examined with qPCR (Table 3). Our qPCR analysis revealed similar induction levels and trends for all these genes as was seen from RNA-seq analyses, indicating that changes in expression found by RNA-seq appear to be accurate.

**Table 3:** qPCR confirmation of select transcripts identified by RNA-sequencing.

Gene ID	Annotations	Log2 FC*-RNA seq	Log2 FC-qRT-PCR
Solyc04g078460	N(4)-(Beta-N-acetylglucosaminyl)-L-asparaginase	4.17	4.77
Solyc03g111400	Xanthine/uracil permease family protein	3.17	3.04
Solyc05g006420	SlRRA1	5.66	3.73
Solyc12g044200	Cc-nbs-lrr resistance protein	−1.97	−0.63
Solyc04g008110	SlHK4	3.87	3.42
Solyc01g108210	Cytochrome P450	4.92	3.86
Solyc02g071220	SlRRA2	4.32	4.24
Solyc12g008900	SlCKX6	8.79	4.08

Transcripts that were identified as cytokinin responsive in 35d leaf samples treated with cytokinin vs. DMSO for 24 h using RNAseq were examined using qPCR. Shown is the log2 fold change calculated from cytokinin vs DMSO for RNAseq and qPCR analyses. FC = fold change.

**Figure 2:**
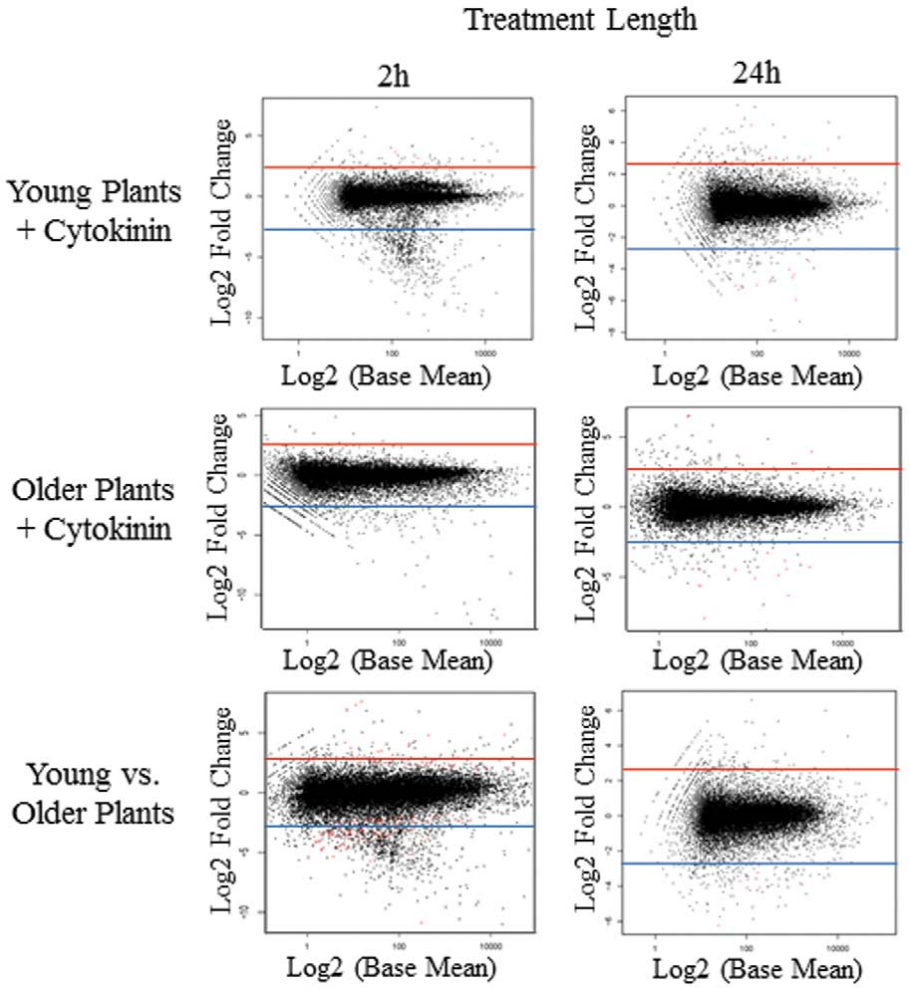
MvA Plots of Leaf Expression Analysis. MvA plots are presented as log2 fold change vs. the log2 base mean for either 2 h or 24 h of treatment. Top shows plots of young (13d) plants treated with cytokinin (5 µM BA) compared to a vehicle control (DMSO). Middle shows plots of older (35d) plants treated with cytokinin (5 µM BA) compared to a vehicle control (DMSO). Bottom shows plots of comparisons between young (13d) and older (35d) plants after only vehicle control (DMSO) treatment. Lines in each graph indicate 2.5 log2 fold change levels, above which transcripts were primarily examined. Dots colored in red represent genes that were identified as differentially expressed by DESeq [90] with BH (Benjamini-Hochberg) adjusted p-values of 0.1 or less in each of the given comparisons.

Overall, using the criteria mentioned above a large number of transcripts was shown to be responsive to the application of exogenous cytokinin (5 µM BA) vs. the solvent vehicle DMSO in both young and older leaves (Table 2). Because of the large number of transcripts that show transcript changes more than 2.5 log2 fold for the different length cytokinin treatments examined, early (2 h) and late (24 h) in leaves of two ages, we present and discuss here a subset of these (Figure 2, Table 2, Table S4, S5, S6) with the rest shown in supplemental data. Since most prior studies of cytokinin response at a transcriptome level in other species like Arabidopsis and rice have focused on and shown a small, but consistent set of transcripts that are induced by cytokinin [10], we have concentrated on reporting the positively cytokinin responsive or induced transcripts here.

**Figure 3:**
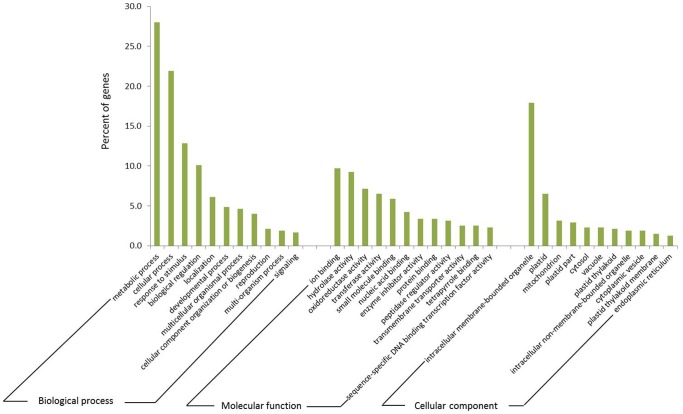
Gene ontology analysis of cytokinin regulated genes in both young and older leaves. The percent of cytokinin regulated genes which belong to each of the major GO categories identified is shown.

In order to have an overall picture of how cytokinin affects gene expression in tomato leaves, we performed gene ontology analysis on the genes identified as cytokinin induced and repressed (Figure 3). Within the biological process class, a large number of cytokinin responsive genes fall into the categories of metabolic process, cellular process, response to stimulus, biological regulation, and developmental process, indicating that cytokinin plays a role in the regulation of cellular metabolism, dealing with external stimulus, and development in plants. Within the molecular function class, many cytokinin responsive genes show binding activity (binding to ions, small molecules, nucleic acids, and proteins), enzyme activity, transporter activity, and transcription factor activity. This demonstrates that cytokinin affects genes that encode proteins with diverse functions such as transcription factor genes that can regulate plant growth and development by activating or repressing their specific target genes. Many of these cytokinin responsive genes encode proteins that are localized in intracellular membrane bounded organelles, plastids, mitochondria, cytosol, and vacuole. The plastid thylakoid localization indicates that a number of cytokinin responsive genes are involved in photosynthesis-related processes.

The gene ontology analysis indicates that a number of cytokinin responsive genes are involved in signaling (Figure 3). A close look at the overall RNA seq data shows that some components of cytokinin signaling pathway such as the cytokinin receptor SlHK4 and the type-A response regulators (SlRRAs) were induced by cytokinin, whereas the type-B RRs were not (Table S7). Several SlCKX genes encoding cytokinin oxidases were also induced by cytokinin (Table S7). It seems that cytokinin treatment has little effect (<2.5 log2 fold) on the expression of histidine phosphotransfer protein encoding genes (Table S7). Overall the cytokinin responsiveness of these cytokinin signaling components mirrors what has been seen in several previous studies [10]. Since hormone crosstalk often occurs, we also looked at whether cytokinin treatment has an effect on the biosynthetic genes of other plant hormones such as auxin and ABA, although most of these genes are not known in tomato. We examined a number of aldehyde oxidases and nitrilases thought to be involved in auxin biosynthesis were detectable but not greatly affected by cytokinin (<2.5 log2 fold, Table S7). An ABA biosynthetic enzyme, the 9-cis-epoxycarotenoid dioxygenase, does not seem to be affected much by cytokinin either, although it might be slightly repressed by cytokinin since the fold change is near or above two fold (Table S7).

### Young Leaves Early Cytokinin Response

We identified more than 700 transcripts that showed transcript change due to an early (2 h) cytokinin treatment in young (13d) tomato leaves (Table 2, Table S4). From this we found 60 genes that were induced at least 2.5 log2 fold by cytokinin 2 h after treatment (Table S4). These genes have diverse functions such as signal transduction, transcriptional regulation, metabolism, transport, and photosynthesis, although several have unknown functions. Within this group of genes there are several that are linked to induction by cytokinin in other species. One of these classes of genes is the type-A response regulators, which have been previously shown to be rapidly induced by cytokinin through different approaches and are almost always in the top set of cytokinin induced genes in transcriptome analyses [3], [8], [9], [10], [32]. We identified four different type-A response regulators that are highly induced, from 3.12–4.12 log2 fold (Table S4). We have designated these as *Solanum lycopersicum* Response Regulator type-As: *Sl*RRA1 to 3, and A6 (Solyc05g006420-SlRRA1, Solyc02g071220-*Sl*RRA2, Solyc10g079600*-Sl*RRA3, and Solyc06g048930*-Sl*RRA6). Two other classes of commonly found cytokinin induced genes were also identified in this sample: two cytokinin oxidases and a cytokinin receptor. The transcripts Solyc01g088160.2 and Solyc04g016430 encoding a cytokinin oxidase were induced 3.7 and 5.4 log2 fold, respectively. Cytokinin oxidase (CKX) is an enzyme which catalyzes the degradation of cytokinin, and it is not surprising to see it induced since if the plant is exposed to excess levels of cytokinin there would be an attempt to break it down using this enzyme [33], [34]. Interestingly it has been reported that reduced expression of the rice cytokinin oxidase gene OsCKX2 can result in increased grain yield, indicating the potential of this gene in crop improvement [35].The transcript Solyc04g008110, a histidine kinase was also induced 2.7 log2 fold, which we verified by qRT-PCR as induced to a similar level (Table 6). This gene, which we have designated *Solanum lycopersicum* Histidine Kinase 4 (*Sl*HK4) encodes the cytokinin receptor most similar to AHK4 in Arabidopsis that has been noted to be induced by cytokinin in several studies.

The four genes that were identified as the most highly induced from 7.4–5.4 log2 fold by cytokinin in young leaves were a CONSTANS-like protein (Solyc07g006630), a UDP-glucuronosyltransferase gene (Solyc12g009930), a peptide transporter gene (Solyc07g008520), and a cytokinin oxidase gene (Solyc04g016430) already discussed. The CONSTANS-like protein (Solyc07g006630) identified has not been assigned any particular function to our knowledge, however, CONSTANS-like proteins (COLs) are known as a group of plant-unique transcription factors which contain a CCT (CONSTANS, CONSTANS-LIKE, and TIMING OF CAB1) domain [36], [37]. Arabidopsis CONSTANS protein was shown to control flowering in response to photoperiod [36], [37]. Tomato is not a photoperiodic plant, and little is known about the tomato COL proteins. Although an Arabidopsis COL gene (At4g39070) was also found up-regulated by cytokinin in CKX1 overexpressing plants [9], how these genes are involved in cytokinin regulated processes remain unknown.

A gene encoding UDP-glucuronosyltransferase (Solyc12g009930) was highly induced by cytokinin as well. Glycosylation is known to play an important role in the regulation of cellular metabolism by altering activity, solubility, and transport of aglycones like plant hormones, secondary metabolites, and xenobiotics [38], [39]. UDP-glucuronosyl-transferases are multi-family enzymes which catalyze the transfer of a glucuronosyl group from a UDP-glucuronic acid to various lipophilic aglycones and are mainly found in insects, fish, and mammals [40]. Glucuronidation enhances polarity and excretability of aglycones and is considered an important mechanism in detoxifying and eliminating lipophilic wastes in the body [40], [41]. Interestingly, overexpression of a pea UDP-glucuronosyltransferase-encoding gene, PsUGT1, resulted in early senescence phenotype in Arabidopsis and reduction of the expression of this gene in alfalfa delayed root emergence and enhanced lateral root development [42]. Since PsUGT1was found to be expressed in regions with active cell division such as root apical meristems [42], leaf primordial and tips of older leaves, it would be interesting to examine whether the cytokinin inducible tomato UDP-glucuronosyltransferase encoding gene plays a role in leaf development.

The third highly induced cytokinin induced gene is a peptide transporter (PTR) gene. Although PTRs have not been previously linked to cytokinin in tomato, s recent study has identified a Medicago gene, LATD/NIP as cytokinin up-regulated in roots which encodes a member of the NRT1/PTR transporter family [43]; it is not known yet whether this gene encodes a nitrate or peptide transporter [43], [44]. The cytokinin induction of the peptide transporter indicates the involvement of cytokinin in the regulation of peptide transport in young tomato leaves; the specific function of this transporter in relation to cytokinin remains to be examined.

A few other interesting genes were also seen as induced by cytokinin in young plants after 2 h of treatment. This includes a few that have some connections to cytokinin or hormone signaling. One of these was surprisingly, a gene encoding a tRNA dimethylallyltransferase (Solyc09g064910), which was induced 4.6 log2 fold. This enzyme catalyzes the isopentenylation of certain tRNAs in bacteria, animals, and plants [45], [46]. In Arabidopsis two genes encoding the tRNA dimethylallyltransferase, *AtIPT2 and AtIPT9* have been identified [45], [47]. Similar to the bacterial *miaA* gene which isopentenylates some tRNAs to synthesize low-level cytokinins [48], [49], these two genes play an indispensable role in the production of cis-zeatin-type cytokinins in plants [46]. Given the fact that the tomato tRNA dimethylallyltransferase was highly induced by cytokinin only in young expanding leaves and that *AtIPT2 and AtIPT9* were more abundant in proliferating tissues [47], it would be interesting to examine the roles of cis-zeatin-type cytokinins in shoot and root apical meristems, leaf primordia, and growing leaves, although no role for cis-zeatin is currently known in Eudicots.

Two more genes which are involved in hormone signaling or hormonal homeostasis were up-regulated by cytokinin as well. *BES1-INTERACTING MYC-LIKE PROTEIN 2* (*BIM2*, Solyc03g114720), a gene encoding a transcription factor has been shown to positively regulate brassinosteroid (BR) signaling along with *BIM1* and *BIM3* [50]. The induction of *BIM2* by cytokinin suggests that there could be crosstalk between cytokinin and BR signaling. The second gene encodes a GH3 family protein which has jasmonate (JA)-amino synthetase activity and adenylyltransferase activity according to the Sol Genomics Network (http://solgenomics.net/). This gene was also induced by cytokinin in older leaves (Table S5). A homolog of this gene in Arabidopsis is JAR1 which has been demonstrated to act as a JA-amino synthetase necessary for the activation of JA for optimal signaling [51], [52]. JAR1 produces JA-Ile which is a key signal for the major jasmonate signaling pathway involving *CORONATINE INSENSITIVE 1* (*COI1*) [53], [54]. The cytokinin responsiveness of the tomato JA-amino synthetase encoding gene in both young and old leaves suggests a link of cytokinin signaling to jasmonate signaling pathway.

Interestingly, four genes involved in photosynthesis were also highly induced by cytokinin (Table 2). Three of them are *LHCB* genes (Solyc10g007690, Solyc06g069730, and Solyc12g011450) which encode chlorophyll a/b binding proteins and the fourth is a photosystem II polypeptide (Solyc07g066310). The induction of these *LHCB* genes supports previous findings that cytokinin can dramatically activate *CAB* promoter activity [55]. Although the role of cytokinin in photosynthesis related processes have been extensively studied [55]–[58], how cytokinin acts in these processes remains unclear. Notably, the photosynthesis-related tomato genes were up-regulated by cytokinin only in young leaves with active cell division, indicating a potential development-dependent regulation of cytokinin on the transcription of these genes. Earlier studies have provided evidence that growing young leaves have a higher content of zeatin-type cytokinins than older leaves [59]. A higher cytokinin level is likely to have a positive effect on photosynthesis by activating *LHCB* genes and other unknown mechanisms, thus provides enough energy sources for fast growing leaves.

We also identified a large number, 669 transcripts that were repressed 2 h after cytokinin treatment (Table 2). We are unsure why there was such an abundance of negatively cytokinin responsive or repressed transcripts. The 100 most highly repressed of these are shown in Table S4 (the rest of these are shown in Table S1) and include an over-representation of genes involved in signaling, defense and stress responses, and protein turnover. Three genes involved in auxin transport and responses (Auxin efflux carrier, ARF4, and SAUR) were down regulated potentially as part of an antagonistic relationship between cytokinin and auxin. Interestingly two cytokinin signaling genes (cytokinin receptor and HPt protein) were also found to be repressed.

### Young Leaves Late Cytokinin Response

We identified nearly 200 transcripts that showed transcript change due to a late (24 h) cytokinin treatment in young (13d) tomato leaves (Table 2, Table S4). About half of these cytokinin responsive transcripts were found to be induced by cytokinin after a 24 h treatment, which is nearly twice as many compared to the 2 h treatment in young tomato leaves (Table 2). The majority of cytokinin induced genes in this longer treatment are transcription factors, signaling genes, or genes involved in hormone metabolism (Table S4). Not surprisingly, there is overlap between the two sets of cytokinin induced genes (2 h and 24 h) in young leaves, which includes several type-A response regulators, the *Sl*HK4 cytokinin receptor, a cytokinin oxidase, and a xanthine/uracil permease family protein. In agreement with the increased number of cytokinin induced genes, several other genes directly linked to cytokinin were also found to be induced. This includes two more type-A response regulators (Solyc03113720 and Solyc10g079700: that we have designated *Sl*RRA5 and *Sl*RRA4, respectively) induced 3.0–3.1 log2 fold and an additional cytokinin oxidase (Solyc12g008900) gene induced 7.9 log2 fold (Table S4).

Several other interesting genes were induced by cytokinin in young plants after the 24 h treatment that may have some connections to cytokinin or hormone signaling. Among these are some transcription factor genes including two NAC (NAM) genes induced 2.8–2.9 log2 fold (Solyc08g077110 and Solyc06g061080), a LOB induced 3.7 log2 fold (Solyc12g100150), an ERF2b induced 3.5 log2 fold (Solyc10g050970), and two WRKY members induced 2.9–3.0 log2 fold (Solyc04g07270 and Solyc08g067360) (Table S4). It has been previously shown that some NAM, such as At4g27410, and LOB domain genes were up-regulated by cytokinin in Arabidopsis [9], [60], [61]. Additionally transient silencing of a tomato *SlNAM* gene resulted in smooth leaflet margins and highly reduced numbers of secondary and intercalary leaflets [62], [63], a feature whose regulation has been linked to cytokinin [4]. Previous work has also shown that a LOB domain gene, *ASYMMETRIC LEAVES 2 LIKE 9* (*ASL9/LBD3*) has cytokinin-dependent expression in both Arabidopsis roots and aerial parts especially leaves as well as being identified as a primary target of the cytokinin signaling pathway [64]. Some LOB domain genes have also been linked to the establishment of leaf polarity [65] and boundary delimitation [66], [67]. Here the two NAM proteins and the LOB domain protein identified as cytokinin inducible are worth further examination to determine if they play a role in cytokinin regulated leaf development in tomato.

It is well known that cytokinin is involved in crosstalk with many other hormones like ethylene, ABA, and gibberellin in a diverse range of processes [68]–[71]. Here we find evidence to further support this with three genes encoding enzymes involved in hormone metabolism that were induced 2.9–3.5 log2 fold by cytokinin. These enzymes include a 1-AMINOCYCLOPROPANE-1-CARBOXYLATE (ACC) OXIDASE-like protein (Solyc11g045520) which catalyzes the final step of ethylene biosynthesis [72], [73], a Cytochrome P450 (Solyc01g108210) with ABA 8′-hydroxylase activity which is a key enzyme involved in ABA catabolism [74], and a Gibberellin 2-oxidase 2 (Solyc07g056670) involved in gibberellin degradation [75]. Previous microarray data from other species identified several genes controlling protein turnover as induced by cytokinin [9]. In our study, two genes regulating protein turnover, which were not responsive to cytokinin after a 2 h treatment, were up-regulated by cytokinin after a 24 h treatment. One encodes a ring finger protein (Solyc06g049030), the other codes for a U-box domain-containing protein (Solyc07g020870). This indicates a possible involvement of cytokinin in regulating protein turnover via these induced genes. Cytokinin has been recently linked to the vacuolar targeting of PIN1, an auxin efflux carrier, for lytic degradation [76], linking cytokinin in the regulation of protein turnover affecting auxin transport if not other processes. There were also a few transcripts that appear connected to stress or defense response that were induced. Three genes encoding LRR receptor-like serine/threonine-protein kinases were induced 2.8–3.0 log2 fold by extended cytokinin treatment. These protein kinases are known to have a link to signaling and defense responses in plants [77].

The 24 h-cytokinin treatment repressed many fewer genes (95) than the short cytokinin treatment, but this number of repressed genes is close to the number (73) found for 35d plants (Table S4). Most genes down-regulated by cytokinin in young leaves seem to be involved in metabolic processes. Interestingly, five genes encoding nodulin-like proteins were repressed as well. In contrast, a gene encoding nodulin-like protein was induced to 3.0 log2 fold by cytokinin 24 h after treatment in older leaves (Table S5). These results suggest a potential differential regulation of these nodulin-like genes by cytokinin in an age-dependent manner.

### Older Leaves Early Cytokinin Response

Only a small number of genes (14; Table S5) were found induced by cytokinin 2 h after treatment in older 35d leaves. The transcript Solyc07g054580 encoding a GH3 family protein and the transcript Solyc04g078460 encoding an asparaginase were induced 3.4–2.9 log2 fold and 2.7–2.8 log2 fold respectively, by cytokinin 2 h after treatment in both young and older tomato leaves. We also identified three purine permease encoding genes (Solyc02g071090, Solyc02g071100, and Solyc02g071080) which were highly induced by cytokinin 2 h after treatment in older tomato leaves. It is known that Arabidopsis purine permeases (AtPUP1 and 2) mediate transport of adenine and possibly cytokinins as well [78], [79]. If purine permeases do function as cytokinin transporters, it could be that exogenous application of cytokinin activates these transporters which in turn transport the extra cytokinin to other parts of the plant.

The 2 h cytokinin treatment resulted in the repression of a large number of transcripts in older leaves (279; Table 2) as seen in young leaves at the early time point. However, the absolute number of induced genes in older leaves (14) is fewer than that of young leaves (60) and the ratio of repressed to induced of older leaves (19∶1) is much greater than that of young leaves (11∶1), indicating that cytokinin may have a greater ability to induce genes in young vs. old tissues. A majority of these genes down-regulated by cytokinin in older leaves are involved in signaling, metabolism, stress and defense responses. We listed only the top 100 most highly repressed transcripts in Table S5 with the rest of them shown in Table S1.

### Older Leaves Late Cytokinin Response

After a 24 h cytokinin treatment, the number of genes (91; Table S5) that showed highly increased transcript level in older leaves is very close to that seen in young leaves (97; Table S4). Six type-A response regulator genes were found highly induced by cytokinin (*Sl*RRA1-6) as seen in young plants. Among the cytokinin induced transcripts are several genes encoding proteins involved in hormone signaling and metabolism. These proteins include the cytokinin receptor (*Sl*HK4), three cytokinin oxidases, two cytochrome P450s (Solyc01g108210 and Solyc04g078900) with abscisic acid 8′-hydroxylase activity, a cytochrome P450 (Solyc02g094860) with steroid hydroxylase activity, a Gibberellin 2-oxidase (Solyc07g061720), two GH3 family proteins, and an adenine phosphoribosyltransferase (APT/APRT)-like protein (Solyc08g079020), that has not been previously linked to cytokinin regulation. APRT (EC 2.4.2.7) catalyzes the conversion of adenine to AMP and has been shown to be able to convert N6-benzyladenine to its nucleotide form in young Arabidopsis plants [80], [81]. If the proposed role of APRTs in the inter-conversion of cytokinins is true, induction of the APRT-like gene by cytokinin shown in the present study may result in the conversion of the active cytokinin nucleobase that was exogenously added to its inactive nucleotide in the leaf, thus regulating the level of active cytokinin.

A few other interesting genes that were induced have potential links to either cytokinin or leaf/cell morphology (Table S5). This includes some transcription factors linked to stress and defense responses that encode a dehydration-responsive family protein, ERF4, and a Heat stress transcription factor. The induction of stress- and defense-related genes by cytokinin has been reported in earlier studies as well [3], [9], [82]. A transcript (Solyc04g080780) coding for BEL1-like homeodomain protein 11 was also induced by cytokinin. A few members of the BEL1-like protein family in Arabidopsis were shown to play roles in leaf morphogenesis by interacting with KNOX homeodomain proteins [83], but little is known about other BEL1-like proteins such as the one identified here. Additionally, two transcripts encoding cell wall-related proteins (Expansin protein, Solyc03g093390 and Pectinesterase, Solyc01g099950) were also found induced by cytokinin, in agreement with previous findings [9], [84].

The extended cytokinin treatment in older leaves repressed around 75genes (Table 2). The most repressed genes encode a seed specific protein (Solyc06g072840), an ubiquitin-conjugating enzyme E2 10 (Solyc03g033410), an F-box family protein (Solyc02g068000), and a thioredoxin H protein (Solyc05g006870). Several nodulin-like protein encoding genes were repressed as well, as seen in young leaves treated by cytokinin for 24 h.

### Comparison of Transcriptome Response to Cytokinin in Young and Older Leaves

**Figure 4:**
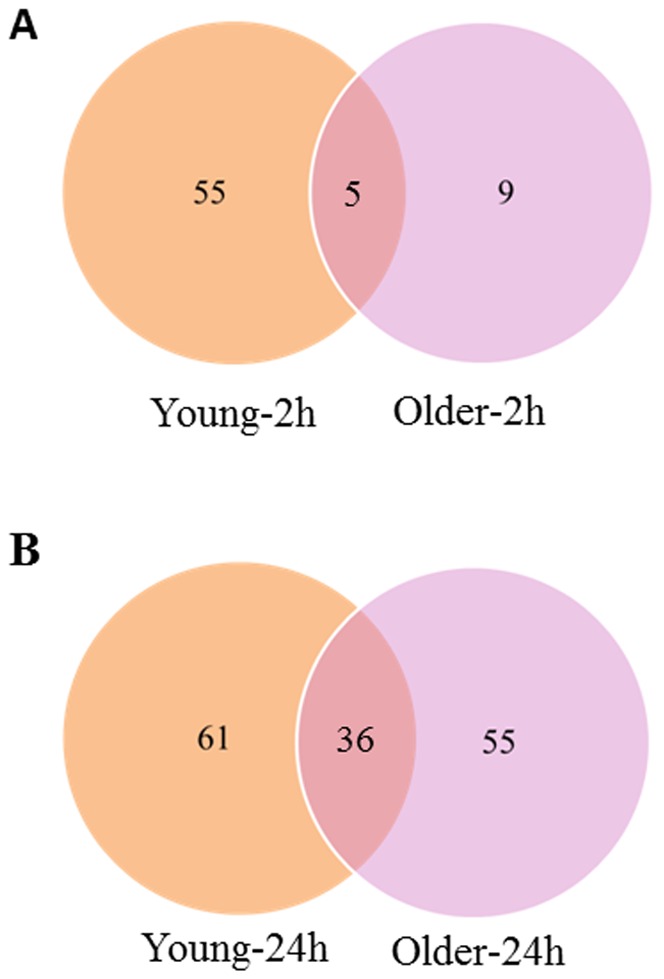
Venn diagram of cytokinin induced genes in young and older leaves. (A) Venn diagram showing number of genes induced by 2 h cytokinin treatment in young and older tomato leaves. (B) Venn diagram showing number of genes induced by 24 h cytokinin treatment in young and older tomato leaves. In both diagrams the number of common genes are shown in the overlapping segment.

Five genes (Solyc01g108210, Solyc04g078460, Solyc07g054580, Solyc09g074490, and Solyc10g079600) were induced by the 2 h cytokinin treatment in both young and older leaves (Table S4, S5, and Figure 4A). This treatment resulted in much more robust response in young leaves compared to older leaves. First, the number of genes induced by cytokinin in young leaves (60) is more than four times that (14) in older leaves (Table 2, Figure 4A). Second, the log2 fold change of young leaves ranges from 2.50 up to 7.34, while that of older leaves ranges from 2.50 to 4.9. Third, more genes know to be involved in cytokinin-related processes of signaling, metabolism, and transport were induced in young leaves (8) compared to older leaves (4).

The 24 h cytokinin treatment induced 36 genes (mainly cytokinin-related genes) in both young and older leaves (Table S4, S5, and Figure 4B) that are more than half of the genes induced either in young or older leaves. Both the number and the range of log2 fold change of cytokinin induced genes in young leaves are comparable to those in older leaves (Table 2, Table S4, S5). However, the number of receptor-like (protein) kinases (7) induced by cytokinin in young leaves is more than three times that (2) in older leaves, indicating a stronger ability of cytokinin to trigger signaling transduction in young leaves. Importantly, the 24 h data indicates that cytokinin is able to induce different genes which fall into the same gene families in young and older leaves.

### Genes Expressed More Abundantly in Young and Older Leaves

Using untreated (DMSO) 2 h data, the number of transcripts (926; Table 2) identified as expressed more abundantly in young leaves is five times as many that (168; Table 2) in older leaves, indicating development-dependent expression of these transcripts. The expression levels of the more abundant transcripts in young leaves ranges from 2.5 to 11.0 log2 fold relative to that in older leaves, in contrast to the range of 2.5 to 7.6 log2 fold for more abundant transcripts in older leaves relative to that in young leaves (Table S6). The abundant transcripts in young leaves include at least three which are cytokinin-related genes, among which one is a type-A response regulator (Solyc11g072330:*Sl*RRA8), one is involved in cytokinin transport (Solyc02g071080: purine permease family protein), and one is cytokinin inducible (Solyc03g115900: chlorophyll a-b binding protein). In older leaves, at least seven cytokinin-related genes were found, among which three encode type-A response regulators (Solyc06g048930: *Sl*RRA6, Solyc02g071220: *Sl*RRA2, Solyc05g006420: *Sl*RRA1), two are cytokinin inducible (Solyc12g011450: chlorophyll a–b binding protein 13, Solyc07g006630: CONSTANS-like protein), and two are involved in cytokinin metabolism (Solyc04g016430: *Sl*CKX5, Solyc09g064910: tRNA dimethylallyltransferase). None of the cytokinin related genes found in young leaves were identified as DE genes, while two (Solyc07g00663 and Solyc09g064910) out of the seven cytokinin related genes found in older leaves were identified as DE genes. Among the top 100 abundant transcripts (the rest of them are shown in Table S2) in young leaves are several genes encoding proteins which function in transcription, translation, cell division, and signal transduction (Table S6). In contrast, the majority of the highly expressed transcripts in older leaves have functions in various metabolic processes. Interestingly, both young and older leaves showed high expression levels of several different signaling genes, such as receptor like kinases indicating that differential types of signaling play vital roles across development.

From the 24 h DMSO treatment data, we also identified a large number of transcripts more abundant in young leaves (198 genes; Table 2) or in older leaves (123 genes; Table S6). In the top 100 abundant transcripts in young leaves (the rest of them are shown in Table S2) there were six chlorophyll a/b binding proteins, four receptor-like kinases, and three UDP-glucosyltransferases. In the top 100 highly expressed transcripts in older leaves there were four cytochrome P450s, five different receptor like kinases, six genes functioning in defense or stress response, and three genes involved in protein degradation.

We also examined the abundant transcripts that were present at 2 h and 24 h of DMSO treatment in each age sample. Although there was not much overlap between lists of abundant transcripts using a log2 fold cutoff, a reduction in the cutoff to log1.5 fold revealed that all abundant transcripts seen at 24 h were also present as abundant transcripts in the 2 h list. Additionally, it is important to note that all 18383 filtered genes used for comparisons were found in both 2 and 24 hour treatment samples in both young and older leaf tissue sample, indicating that these samples are largely similar.

## Materials and Methods

### Plant Materials and Growth Conditions

The tomato cultivar Micro-Tom was used for all experiments. Plants were grown in Sunshine Mix #8 soil under a 16 h light/8 h dark photoperiod at 150 µE, with a 26°C day(light), 22°C night (dark) temperature.

### Cytokinin Treatment and RNA Extraction

In each sample treatment six leaves each from different individual plants were excised. For both 13d and 35d old plants only the apical most fully expanded leaves were collected in this manner. In 13d plants these were the only true leaves that were fully expanded and present. The excised leaves were placed in water, and gently shaken for 2 h prior to treatment with cytokinin 5 µM benzyladenine (BA) and the solvent control DMSO for 2 h or 24 h. At the end of treatment leaves were patted dry then immediately flash-frozen in liquid nitrogen [7], [85]. RNA was subsequently extracted using Qiagen RNeasy Kit according to the manufacturer’s instructions.

### Library Preparation and Sequencing

Messenger RNA was isolated with polyA selection and constructed into paired end sequencing libraries with an insert size of 180 bp with the TruSeq RNA sample preparation protocol from Illumina (San Diego, CA).

Paired-end sequencing was performed on 16 samples on the Illumina HiSeq 2000 platform, generating 131,158,386 2×50 bp read pairs. Additionally, 60,180,592 1×54 bp single-end reads were generated on the Illumina GAIIX platform to attain adequate read counts for each sample for assessing differential expression. In total, over 16.4 Gbp were sequenced for *de novo* assembly and differential expression analysis. Raw sequence data is available for download at NCBI Sequence Read Archive under the accession (currently awaiting SRP # assignment).

### Assembly

Paired-end sequences from 16 samples were pooled together to construct a *de novo* tomato leaf transcriptome assembly. Reads passing initial Illumina filters were further trimmed with the FASTX-Toolkit [86] at the 3′ end with a quality score threshold of Q15. Reads were first assembled with ABySS (v1.2.6) [87] with a kmer sweep of select kmers from 25 to 50 and scaffolding enabled. Gaps in the assembly were closed with GapCloser (v1.10, SOAP package) [88]. Contigs from the kmer-sweep were pooled and deredundified with CD-HIT-EST (v4.5.4) [89]. An overlap-layout-consensus assembly from these contigs, or synthetic ESTs, was created with MIRA (v3.2.1) [90] operated in Sanger EST mode. The final assembly contained 28,606 synthetic ESTs and was used as a reference for subsequent gene expression analysis.

### Expression Analysis with Custom Transcriptome Reference

The 3′-trimmed reads used in *de novo* assembly and additional single-end sequences were aligned to the final assembly with BWA with default settings (v0.5.9) [30]. Gene expression was quantified as the total number of reads for each sample that uniquely aligned to the reference, binned by transcript. Twelve comparisons wherein one variable changed were performed to elucidate the transcripts differentially expressed with age (13 and 35 days), treatment (cytokinin and control vector), and treatment length (2 h and 24 h). To perform robust analyses, we only considered transcripts that were covered by at least 2 reads per million in at least 2 samples in any given comparison; this reduced the number of transcripts assessed from 28,606 to 18,838. Differential expression analysis of these, per-sample read counts was performed with the negative binomial test in DESeq [91]. Genes were identified as differentially expressed if they had an adjusted (Benjamini-Hochberg False Discovery Rate (FDR) method for multiple testing correction) p-value of 0.1 or less. These transcripts were annotated against the International Tomato Annotation Group (ITAG) Solanum lycopersicum protein reference version 2.3 reference with BLASTx [92].

### Gene Ontology Analysis

The functional annotation software Blast2go (http://www.blast2go.com/b2ghome) was used to conduct gene ontology analysis of the cytokinin responsive genes in this study. The major GO categories to which the cytokinin responsive genes belong were determined after the genes were subject to BLAST, mapping, and annotation. Results were presented as a bar chart showing the percent of genes belonging to each GO category identified.

### qPCR Analysis

To synthesize cDNA, 500 ng of the total RNA, the same as isolated for RNA-seq analysis, was used for each sample in the reverse transcription with Quanta qScript cDNA supermix. The first strand of cDNA was diluted 50 times before it was used in the qRT-PCR. qRT-PCR was performed with the SYBR-Green chemistry in an Eppendorf Mastercycler ep realplex with gene specific primers (Table S3). Each reaction contains 9 µL of SYBR-Green supermix, 5 µL of cDNA template, and 3 µL of forward and reverse primers (4 µM). The qRT-PCR program consists of one cycle at 95°C for 15 sec, followed by 40 cycles of 15 sec at 95°C, 45 sec at 57°C, and 25 sec or 40 sec at 68°C. The relative expression data used in the table represent means ± SE of two biological replicates. All samples are compared to the control gene TIP41 [93].

## Supporting Information

Table S1 **Transcripts repressed by 2 h cytokinin treatment in both young and older leaves.** The top 100 most highly repressed transcripts were shown in Table S4 and Table S5.
(XLSX)

Table S2 **Transcripts identified as more abundant (2.5 log2 fold greater than the other age sample at that treatment time) in control leaf samples.** The top 100 transcripts were shown in Table S6.
(XLSX)

Table S3 **Primer sequences used for qPCR validation of select transcripts identified from RNA sequencing.**
(XLSX)

Table S4 **Transcripts identified as up-regulated or repressed 2.5 log2 fold by cytokinin in young leaves.** The 2 h cytokinin treatment repressed a large number of transcripts and only the top 100 transcripts identified as most highly repressed by cytokinin were listed here (the rest of these are shown in Table S1). FC = fold change.
(XLSX)

Table S5 **Transcripts identified as up-regulated or repressed 2.5 log2 fold by cytokinin in older leaves.** The 2 h cytokinin treatment repressed a large number of transcripts and only the top 100 transcripts identified as most highly repressed by cytokinin were listed here (the rest of these are shown in Table S1). FC = fold change.
(XLSX)

Table S6 **Transcripts identified as more abundant (2.5 log2 fold greater than the other age sample at that treatment time) in control leaf samples.** Only the top 100 transcripts were listed in the table. FC = fold change.
(XLSX)

Table S7 **Response to cytokinin of transcripts that are involved in hormone signaling and metabolism.** These transcripts listed include those that are involved in cytokinin signaling and metabolism, auxin biosynthesis and ABA biosynthesis.
(XLSX)
