# Correction: Transcriptome Analysis of Cytokinin Response in Tomato Leaves

**DOI:** 10.1371/annotation/58de6bf0-996f-418c-8970-13bbae3ddc20

**Published:** 2013-08-08

**Authors:** Xiuling Shi, Sarika Gupta, Ingrid E. Lindquist, Connor T. Cameron, Joann Mudge, Aaron M. Rashotte

Two grants were incorrectly omitted from the Funding statement. The following sentence should be added to the current Funding statement: "This project was supported by the National Institute of Health through grants from the National Center for Research Resources (5P20RR016480-12) and the National Institute of General Medical Sciences (8P20GM103451-12)." 

